# Preventive Immunomodulatory Effects of Doxycycline and Hyperbaric Oxygen Therapy on Serum Tumor Necrosis Factor‐*α* and Interleukin‐6 Levels in a Rat Model of Malaria

**DOI:** 10.1155/japr/7020257

**Published:** 2026-10-27

**Authors:** Risma Risma, I Made Prasetya Kurniawan, Djatiwidodo Edi Pratiknya, Evelyn Tesalonika Marietta, Edwin Timotius Martua, Jovita Nathania

**Affiliations:** ^1^ Department of Parasitology Faculty of Medicine Hang Tuah University Surabaya East Java Indonesia hangtuah.ac.id; ^2^ Department of Marine Health Faculty of Medicine Hang Tuah University Surabaya East Java Indonesia hangtuah.ac.id; ^3^ Undergraduate Medical Study Program Faculty of Medicine Hang Tuah University Surabaya East Java Indonesia hangtuah.ac.id; ^4^ Indonesian Navy Headquarters East Jakarta Jakarta Indonesia

**Keywords:** doxycycline, hyperbaric oxygen therapy, IL-6, malaria prophylaxis, TNF-*α*

## Abstract

**Background:**

Malaria remains a major global health problem causing morbidity and mortality. Although doxycycline is widely used as an effective malaria chemoprophylactic agent, its use is limited in certain populations (doxycycline should not be given to children under 8 years of age and pregnant women). These limitations, together with the growing challenge of antimalarial drug resistance, necessitate the exploration of adjunctive and alternative prophylactic strategies. Hyperbaric oxygen therapy (HBOT), which involves inhalation of 100% oxygen under increased atmospheric pressure, has demonstrated immunomodulatory and anti‐inflammatory effects in various infectious diseases.

**Objective:**

This study is aimed at evaluating the preventive immunomodulatory effects of doxycycline and HBOT, alone or in combination, on the serum levels of proinflammatory cytokines tumor necrosis factor‐*α* (TNF‐*α*) and interleukin‐6 (IL‐6) in a *Plasmodium berghei* rat model.

**Methods:**

An in vivo experimental study was conducted using 24 female BALB/c mice (7–10 weeks old), randomly assigned into four groups: control group (CG), doxycycline group (DG), hyperbaric oxygen therapy group (HG), and combined doxycycline–hyperbaric oxygen therapy group (DHG). All groups were infected with *P*. *berghei* ANKA. Serum TNF‐*α* and IL‐6 levels were measured and compared among the groups.

**Results:**

Significant differences in TNF‐*α* (*p* = 0.012), IL‐6 (*p* = 0.010), and parasitemia (*p* = 0.001) were observed among the four groups. TNF‐*α* levels were significantly lower in DG, HG, and DHG than in CG (*p* = 0.037, *p* = 0.004, and *p* = 0.004, respectively), whereas no significant differences were observed among the treatment groups. IL‐6 was significantly lower in HG and DHG than in CG (both *p* = 0.004), whereas the difference between DG and CG was not statistically significant (*p* = 0.150). All treatment groups showed significantly lower parasitemia than CG (all *p* = 0.001). HBOT and DHG also showed lower parasitemia than DG (*p* = 0.020 and *p* = 0.006, respectively), whereas no significant difference was observed between HG and DHG (*p* = 0.952).

**Conclusion:**

Both doxycycline and HBOT exert immunomodulatory effects by reducing TNF‐*α* and IL‐6 levels in experimental malaria. The present findings suggest that doxycycline and HBOT may modulate systemic inflammatory responses during experimental malaria. Combination of doxycycline and HBOT shows a favorable anti‐inflammatory profile against malaria‐associated inflammation.

## 1. Introduction

Malaria remains a major global health problem, particularly in tropical and subtropical regions, where it contributes substantially to morbidity and mortality. The disease is caused by protozoan parasites of the genus *Plasmodium* that invade erythrocytes and is transmitted between humans through the bite of an infected female *Anopheles* mosquito [1]. The disease has a complex biological cycle involving mosquito, hepatic, and erythrocytic stages, and its clinical manifestations range from uncomplicated febrile illness to severe anemia, respiratory distress, multiorgan dysfunction, and cerebral malaria. The severity of disease is determined not only by parasite burden but also by the magnitude and regulation of the host immune response [1, 2]. The four principal species responsible for human malaria are *Plasmodium malariae*, *Plasmodium ovale*, *Plasmodium falciparum*, and *Plasmodium vivax*. Among these, *P*. *falciparum* is associated with the most severe clinical manifestations and can be fatal if not promptly and adequately treated [2, 3].

*P*. *falciparum* is the deadliest malaria parasite and remains the leading cause of malaria‐related morbidity and mortality worldwide. According to the 2023 Global Burden of Disease report, more than 263 million malaria cases were reported globally, resulting in approximately 597,000 deaths, predominantly in sub‐Saharan Africa and Southeast Asia [2]. Malaria is also highly prevalent in Indonesia, an endemic country that reported 443,530 cases, with approximately 89% of confirmed cases occurring in Papua Province. The widespread resistance of *P*. *falciparum* to chloroquine and other antimalarial agents continues to influence the selection of effective malaria chemoprophylactic regimens [2, 4]. The discovery of artemisinin and the subsequent development of artemisinin‐based combination therapies (ACTs) represented a major therapeutic breakthrough and remains central to contemporary malaria management [2, 3]. Nevertheless, the emergence and geographic expansion of partial artemisinin resistance, together with resistance to partner drugs, threaten the durability of current treatment strategies [2].

Doxycycline is an antibiotic widely used for malaria prophylaxis, with reported efficacy ranging from 92% to 100%. However, its use is contraindicated in children under 8 years of age and in pregnant women, as tetracycline‐class antibiotics are known to cross the placenta and enter the fetal circulation and amniotic fluid. These limitations, together with the increasing prevalence of multidrug‐resistant *P*. *falciparum*, underscore the need to explore alternative and adjunctive prophylactic strategies to reduce the risk of failure associated with current antimalarial regimens [3].

Doxycycline is a well‐established antimalarial adjunct, commonly used for prophylaxis and combination therapy. Beyond its antiparasitic effects through inhibition of apicoplast protein synthesis, doxycycline also has the ability to modulate the immune system and reduce inflammation. It can lower the levels of certain inflammatory chemicals in the body, like tumor necrosis factor‐*α* (TNF‐*α*) and interleukin‐6 (IL‐6). New research indicates that doxycycline might help reduce widespread inflammation caused by malaria. However, its immunomodulatory efficacy in comparison with nonpharmacological interventions remains insufficiently explored [3]. TNF‐*α* and IL‐6 are key mediators in malaria pathogenesis. TNF‐*α* promotes macrophage activation and nitric oxide production, facilitating parasite killing; however, sustained overexpression is associated with endothelial dysfunction, blood–brain barrier disruption, and severe malaria manifestations. Similarly, IL‐6 is involved in acute‐phase responses and immune regulation, yet elevated IL‐6 levels correlate with disease severity and poor outcomes in experimental and human malaria. Therefore, therapeutic strategies that can modulate excessive inflammatory responses without compromising antiparasitic immunity are of significant interest [1, 3].

Hyperbaric oxygen therapy (HBOT) is the latest breakthrough therapy that has many clinical benefits in various diseases. HBOT is still undergoing further research on its very broad benefits. HBOT is a treatment where a person breathes 100% oxygen in a chamber that has higher atmospheric pressure than normal and has emerged as a promising adjunctive therapy for infectious and inflammatory diseases. HBOT increases tissue oxygen tension, enhances reactive oxygen species (ROS)–mediated microbial killing, and modulates immune responses. Experimental studies in rat malaria models have demonstrated that HBOT can reduce parasitemia, down‐regulate proinflammatory cytokines including TNF‐*α* and IL‐6, and mitigate inflammatory cell sequestration in target organs. In addition, HBOT has been found to have a systemic anti‐inflammatory effect by lowering the levels of IL‐6 and TNF‐*α* in different types of infections and inflammatory diseases [4, 5].

HBOT has been widely used in various medical conditions, both acute and chronic. Some indications for this therapy that have been recognized by various medical associations include carbon monoxide poisoning, decompression sickness, nonhealing wounds in diabetics, severe soft tissue infections, and radiation injuries. The primary way HBOT works is by raising the level of oxygen in the tissues, which significantly improves how well the body uses oxygen. This enhanced oxygen supply helps fibroblasts function better and promotes the growth of new blood vessels [6].

Although both doxycycline and HBOT have demonstrated anti‐inflammatory properties, direct comparative studies evaluating their effects on key proinflammatory cytokines, such as TNF‐*α* and IL‐6, in malaria are currently lacking. Understanding the differential immunomodulatory effects of pharmacological and oxygen‐based interventions may provide valuable insights into adjunctive therapeutic strategies for malaria. Therefore, this study is aimed at comparing TNF‐*α* and IL‐6 levels in a *Plasmodium berghei* rat model to evaluate the immunomodulatory effects of doxycycline alone and in combination with HBOT, with the expectation that these findings may contribute to the development of novel approaches for controlling malaria‐associated inflammation.

## 2. Materials and Methods

### 2.1. Study Design

This was an in vivo experimental study using a randomized controlled group design with posttest‐only analysis. The experiment was conducted at the Comprehensive Molecular Hyperbaric Laboratory, Faculty of Medicine, Hang Tuah University, Surabaya, Indonesia.

A total of 24 female BALB/c mice (7–10 weeks old) were used in this study. The animals were obtained from PUSVETMA Surabaya and were acclimatized for 1 week prior to experimentation under standard laboratory conditions. The mice were randomly allocated into four experimental groups (*n* = 6 per group): (1) the control group (CG) was infected with *P*. *berghei* ANKA and did not receive doxycycline or HBOT, (2) the doxycycline group (DG) was infected with *P*. *berghei* ANKA and administered doxycycline at a prophylactic dose of 25 mg/kg body weight, (3) the hyperbaric oxygen therapy group (HG) was infected with *P*. *berghei* ANKA and received HBOT alone, and (4) the combined doxycycline–hyperbaric oxygen therapy group (DHG) was infected with *P*. *berghei* ANKA and treated with a combination of doxycycline (25 mg/kg body weight) and HBOT. All prophylactic interventions were administered for three consecutive days prior to cytokine measurement.

### 2.2. Donor Mice Creation

Five donor mice were used for parasite propagation. Each mouse was inoculated with 200 *μ*L of frozen blood containing *P*. *berghei* ANKA. When parasitemia exceeded 20%, the donor mice were euthanized. Blood was collected by cardiac puncture and subsequently transferred into the peritoneal cavity of the experimental mice for infection. All mice in the experimental groups were infected with 200 *μ*L of infected blood containing approximately 1 × 10^7^ parasitized erythrocytes.

### 2.3. Data Analysis

Serum levels of TNF‐*α* and IL‐6 were measured using a sandwich enzyme‐linked immunosorbent assay (ELISA) kit (Cat. No. E0117Mo, BT Laboratory, Shanghai, China) according to the manufacturer′s instructions. The sensitivity limit is 2.49 pg/L for TNF‐*α* and 1.51 pg/ml for IL‐6. The Kruskal–Wallis test was used to compare TNF‐*α* and IL‐6 levels among the four experimental groups, followed by the Mann–Whitney *U* test for pairwise post hoc comparisons. For parasitemia, one‐way analysis of variance (ANOVA) was used to compare the four experimental groups, followed by Tukey′s honestly significant difference (HSD) test for pairwise comparisons. A *p* value < 0.05 was considered statistically significant.

## 3. Results

Table 1 showed that TNF‐*α* levels differed significantly among groups (*p* = 0.012). The CG exhibited the highest TNF‐*α* concentrations, with a mean value of 312.96 pg/mL. In contrast, all treatment groups showed lower TNF‐*α* levels. The DG demonstrated a mean TNF‐*α* level of 212.63 pg/mL, whereas the HG and the combined therapy group showed further reductions, with mean values of 180.27 pg/mL and 195.28 pg/mL, respectively.

**Table 1:** Descriptive statistics of serum tumor necrosis factor‐*α* (TNF‐*α*) levels (pg/mL) among *P*. *berghei*–infected mice in the control, doxycycline, hyperbaric oxygen therapy (HBOT), and combined doxycycline–HBOT groups.

Group	Tumor necrosis factor‐*α*						*p*
	Median	Mean	Interquartile		Var	SD	
			Q1	Q3			
CG (*n* = 6)	298.76	312.96	267.72	401.63	2175.67	46.64	0.012 ^∗^
DG (*n* = 6)	196.20	212.63	136.85	333.46	5846.93	76.46	
HG (*n* = 6)	184.33	180.27	160.59	198.94	199.73	14.13	
DHG (*n* = 6)	194.37	195.28	133.81	242.77	1731.11	41.60	

^∗^Statistical analysis using Kruskal–Wallis test.

Abbreviations: CG, control group; DG, doxycycline group; DHG, combined doxycycline–hyperbaric oxygen therapy group; HG, hyperbaric oxygen therapy group; Max, maximum; Min, minimum; SD, standard deviation; Var, variance.

Significant differences in IL‐6 levels were also observed among the study groups (*p* = 0.010), as summarized in Table 2. The highest IL‐6 concentration was detected in the CG, with a mean value of 235.03 pg/mL. All intervention groups demonstrated lower IL‐6 levels, with mean values of 179.83 pg/mL in the DG, 151.42 pg/mL in the HG, and 132.80 pg/mL in the combined therapy group.

**Table 2:** Descriptive statistics of serum interleukin‐6 (IL‐6) levels (pg/mL) among *P*. *berghei*–infected mice in the control, doxycycline, hyperbaric oxygen therapy (HBOT), and combined doxycycline–HBOT groups.

Group	Interleukin‐6						*p*
	Median	Mean	Interquartile		Var	SD	
			Q1	Q3			
CG (*n* = 6)	233.03	235.03	200.34	272.43	948.82	30.80	0.010 ^∗^
DG (*n* = 6)	142.86	179.83	137.49	274.74	3830.13	61.88	
HG (*n* = 6)	153.07	151.42	122.85	172.58	370.58	370.55	
DHG (*n* = 6)	139.38	132.80	112.85	149.38	230.83	15.19	

^∗^Statistical analysis using Kruskal–Wallis test.

Abbreviations: CG, control group; DG, doxycycline group; DHG, combined doxycycline–hyperbaric oxygen therapy group; HG, hyperbaric oxygen therapy group; Max, maximum; Min, minimum; SD, standard deviation; Var, variance.

The Mann–Whitney test demonstrated significant differences in TNF‐*α* levels between the CG and both the DG (*p* = 0.037), the HG (*p* = 0.004), and the DHG (*p* = 0.004). In contrast, no significant differences in TNF‐*α* levels were observed between the DG and HG (*p* = 1.000), DG and DHG (*p* = 0.937), or HG and DHG (*p* = 0.470). These findings indicate that all treatment groups differed significantly from the CG, whereas the three therapeutic approaches did not differ significantly from one another in terms of TNF‐*α* levels, as presented in Table 3.

**Table 3:** Mann–Whitney test of tumor necrosis factor‐*α* (TNF‐*α*) levels among *P*. *berghei*–infected mice in the control, doxycycline, hyperbaric oxygen therapy (HBOT), and combined doxycycline–HBOT groups.

Tumor necrosis factor‐*α*				
Experimental group	CG	DG	HG	DHG
CG	—	0.037	0.004	0.004
DG	0.037	—	1.000	0.937
HG	0.004	1.000	—	0.470
DHG	0.004	0.937	0.470	—

Abbreviations: CG, control group; DG, doxycycline group; DHG, combined doxycycline–hyperbaric oxygen therapy group; HG, hyperbaric oxygen therapy group.

The Mann–Whitney test revealed significant differences in IL‐6 levels between the CG and the HG (*p* = 0.004) as well as between the CG and the DHG (*p* = 0.004). However, the difference between the CG and the DG was not statistically significant (*p* = 0.150). No significant differences were observed among the treatment groups, including between DG and HG (*p* = 0.936), DG and DHG (*p* = 0.261), or HG and DHG (*p* = 0.078), as presented in Table 4. These findings indicate that HBOT, either alone or in combination with doxycycline, was associated with a significant difference in IL‐6 levels compared with the CG, whereas doxycycline alone did not produce a statistically significant difference.

**Table 4:** Mann–Whitney test of interleukin‐6 (IL‐6) levels among *P*. *berghei*–infected mice in the control, doxycycline, hyperbaric oxygen therapy (HBOT), and combined doxycycline–HBOT groups.

Interleukin‐6				
Experimental group	CG	DG	HG	DHG
CG	—	0.150	0.004	0.004
DG	0.150	—	0.936	0.261
HG	0.004	0.936	—	0.078
DHG	0.004	0.261	0.078	—

Abbreviations: CG, control group; DG, doxycycline group; DHG, combined doxycycline–hyperbaric oxygen therapy group; HG, hyperbaric oxygen therapy group.

One‐way ANOVA demonstrated a statistically significant difference in parasitemia among the four experimental groups (*p* = 0.001). The CG had the highest median and mean parasitemia values (37.50 and 38.53, respectively), followed by the DG (24.75 and 26.00), the HG (17.75 and 17.81), and the DHG, which had the lowest values (16.05 and 16.48), as presented in Table 5. These findings indicate that treatment with doxycycline, HBOT, or their combination was associated with lower parasitemia compared with the control group, with the DHG showing the lowest parasitemia levels.

**Table 5:** Distribution data of parasitemia.

Group	Median	Mean	Var	SD	*p*
CG (*n* = 6)	37.50	38.53	18.53	4.30	0.001 ^∗^
DG (*n* = 6)	24.75	26.00	42.94	6.55	
HG (*n* = 6)	17.75	17.81	5.74	2.39	
DHG (*n* = 6)	16.05	16.48	9.85	3.13	

^∗^Statistical analysis using one‐way ANOVA.

Abbreviations: CG, control group; DG, doxycycline group; DHG, combined doxycycline–hyperbaric oxygen therapy group; HG, hyperbaric oxygen therapy group; SD, standard deviation; Var, variance.

The Tukey HSD post hoc analysis demonstrated statistically significant differences in parasitemia levels between the CG and all treatment groups, including the DG, HG, and DHG (all *p* = 0.001). Significant differences were also observed between DG and HG (*p* = 0.020) and between DG and DHG (*p* = 0.006). In contrast, no significant difference was found between HG and DHG (*p* = 0.952), as presented in Table 6. These findings indicate that all treatment groups had significantly lower parasitemia levels than the CG, whereas HBOT alone and the DHG produced comparable effects on parasitemia.

**Table 6:** Tukey HSD test of parasitemia levels among *P*. *berghei*–infected mice in the control, doxycycline, hyperbaric oxygen therapy (HBOT), and combined doxycycline–HBOT groups.

Interleukin‐6				
Experimental group	CG	DG	HG	DHG
CG	—	0.001	0.001	0.001
DG	0.001	—	0.020	0.006
HG	0.001	0.020	—	0.952
DHG	0.001	0.006	0.952	—

Abbreviations: CG, control group; DG, doxycycline group; DHG, combined doxycycline–hyperbaric oxygen therapy group; HG, hyperbaric oxygen therapy group.

## 4. Discussion

The present study demonstrates that doxycycline and HBOT, administered either alone or in combination, were associated with lower systemic inflammatory cytokine levels in *P*. *berghei*–infected mice. TNF‐*α* differed significantly among the four experimental groups (*p* = 0.012), with the highest mean concentration observed in the untreated CG (312.96 pg/mL), compared with 212.63 pg/mL in the DG, 180.27 pg/mL in the HG, and 195.28 pg/mL in the combined group. Similarly, IL‐6 differed significantly among groups (*p* = 0.010), with the highest concentration in the CG (235.03 pg/mL) and lower concentrations in the DG (179.83 pg/mL), HG (151.42 pg/mL), and combined groups (132.80 pg/mL). These findings indicate that the interventions were associated with attenuation of systemic inflammatory responses; however, the biological significance of this reduction should be interpreted in relation to parasite burden and the dual role of inflammatory responses in malaria.

TNF‐*α* is a major proinflammatory cytokine produced by monocytes, macrophages, and T cells in response to parasitic infections such as *Plasmodium* spp. In the context of malaria, TNF‐*α* has a dual role both as a mediator of immune protection against parasites and as a contributor to severe complications, particularly in cerebral malaria. In the early stages of malaria infection, TNF‐*α* functions as part of the innate immune response. TNF‐*α* is produced in response to activation of toll‐like receptors (TLRs) by parasite components such as glycosylphosphatidylinositol (GPI) and parasite DNA. TNF‐*α* enhances phagocytic activity, triggers ROS production, and helps control parasitemia through macrophage activation and neutrophil recruitment to the site of infection [7].

However, excessive TNF‐*α* production can also trigger the pathogenesis of severe malaria. TNF‐*α* increases the levels of ICAM‐1 and VCAM‐1 (adhesion molecules that can be expressed on vascular endothelium), which facilitates the adhesion of *P*. *falciparum*–infected erythrocytes to the blood vessel wall. This causes microvascular obstruction, tissue hypoxia, and leads to cerebral malaria and multiple organ failure [8]. TNF‐*α* also triggers the production of prostaglandin E2 (PGE2) in the hypothalamus, causing fever, which is one of the typical symptoms of malaria. In addition, TNF‐*α* contributes to erythrocyte lysis and hepatocellular dysfunction and can cause increased capillary permeability and plasma leakage, worsening the patient′s clinical condition [9]. This is in line with this study; the results presented in Table 1 show that the average group receiving doxycycline and HBOT (DG, HG, and DHG) had a trend of much lower TNF‐*α* levels compared with the CG that did not receive therapy.

Research has found that higher levels of plasma TNF‐*α* are linked to more severe cases of malaria. Patients who had severe malaria showed higher TNF‐*α* levels than those with mild or moderate malaria [10]. TNF‐*α* is an important mediator of the host response to malaria. Its early production contributes to parasite control through activation of innate immune cells, whereas excessive or sustained TNF‐*α* signaling can promote endothelial activation, leukocyte recruitment, vascular dysfunction, and tissue injury. Therefore, a reduction in TNF‐*α* should not simply be interpreted as evidence of improved immune function. Rather, the therapeutic objective is likely to achieve an appropriate inflammatory balance that preserves antiparasitic activity while limiting inflammation‐associated tissue damage. Clinical studies have consistently associated increased TNF‐*α* concentrations with malaria severity, supporting the relevance of TNF‐*α* as a marker of excessive inflammatory activation [7, 10].

Studies in the ANKA mouse model of *P*. *berghei* infection also show that TNF‐*α* plays a key role in mortality through induction of systemic inflammatory responses and brain injury. A previous study reported that HBOT in mice infected with *P*. *berghei* ANKA can reduce the expression of adhesion molecules ICAM‐1 and transcription factor HIF‐1*α* in brain endothelial cells. Although TNF‐*α* was not measured directly, the decrease in ICAM‐1, which is a downstream target of TNF‐*α*, indicates that HBOT can indirectly reduce TNF‐*α* activity. Another previous study found that HBOT can inhibit the hypoxic response and kininurenin production in mice with experimental cerebral malaria. This contributes to protection against brain damage and decreased inflammation, which most likely involves modulation of TNF‐*α* [4, 11].

The relationship between IL‐6 and parasite burden further supports an integrated interpretation of the findings. IL‐6 is induced during malaria infection and has been associated with parasite density and disease severity in clinical studies. A systematic review and meta‐analysis reported significantly increased IL‐6 concentrations in malaria and an association between IL‐6 levels and disease severity [12]. In the present study, the untreated group had both the highest IL‐6 concentration and the highest parasitemia, whereas the HBOT and combined groups exhibited lower values for both parameters. This parallel reduction suggests that attenuation of parasite‐associated inflammatory stimulation may contribute to the lower IL‐6 concentrations.

IL‐6 is not constitutively produced but is rapidly induced during infection. When the *Plasmodium* spp. infects red blood cells, it releases certain molecules called pathogen‐associated molecular patterns (PAMPs), such as GPI‐anchor (GPI) and hemozoin crystals (malarial pigment). Innate immune cells (mainly macrophages and monocytes) detect these molecules through specific receptors such as TLRs (TLR2 and TLR4). Activation of this receptor triggers a cascade of intracellular signals (via the NF‐*κ*B pathway) that directs the cell nucleus to produce and release the cytokine IL‐6 into the bloodstream. IL‐6 signals the liver to produce acute phase proteins (such as C‐reactive protein) that help in opsonizing (signaling) parasites to make them easier to eat by phagocytic cells [11, 12].

Excessive IL‐6 levels stimulate the production of hepcidin, a hormone that inhibits iron absorption. As a result, the production of new red blood cells (erythropoiesis) is disrupted, causing anemia and damage to the blood–brain barrier, leading to cerebral malaria and multiple organ failure. Although IL‐6 is a defense cytokine, very high levels can cause immune exhaustion, which actually weakens the body′s ability to reduce parasitemia and indirectly increases parasite activity [12, 13].

A previous study through a systematic meta‐analysis showed that serum IL‐6 levels significantly correlated with parasite density (parasitemia) and clinical severity in malaria patients. IL‐6 acts as a major proinflammatory cytokine triggered by the release of parasite products such as hemozoin through the NF‐*κ*B signaling pathway. Although IL‐6 is required to initiate the acute phase response, excessive production actually facilitates an environment that supports parasite replication and vascular damage [12].

According to a previous study in Figure 1, IL‐6 is a major stimulator of hepcidin production in the liver. High IL‐6 levels trigger hyperhepcidinemia, which blocks iron in macrophages. This initially aims to “starve” the parasite, but if chronic, it actually impairs host erythropoiesis and the effectiveness of parasite clearance. Decreasing IL‐6 helps normalize this pathway, thereby enhancing the capacity of the cellular immune response to suppress parasite burden [14].

**Figure 1:**
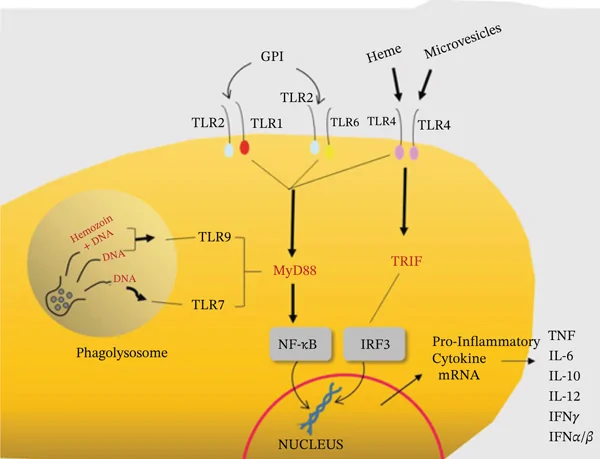
Toll‐like receptor pathway [14].

Furthermore, a study in patients with severe malaria demonstrated that interventions that reduce proinflammatory cytokines, including IL‐6, can halt the “cytokine storm” that often accompanies high parasite burdens. This reduction in IL‐6 activity reduces the overactivation of the STAT3 pathway, which indirectly increases the efficiency of antibody opsonization of infected red blood cells [15]. Modulating IL‐6 to physiological levels prevents the phenomenon of immune exhaustion, allowing T and B cells to work more optimally in gradually reducing parasitemia levels without triggering systemic organ damage [13]. This is in line with this study; the results presented in Table 2 show that the average group receiving doxycycline and HBOT (DG, HG, and DHG) had a trend of much lower IL‐6 levels compared with the CG that did not receive therapy.

HBOT is a treatment where pure oxygen is given at a higher pressure than normal, and it has been found to help reduce inflammation by changing how certain molecules work in the body. One main way HBOT lowers levels of TNF‐*α* and IL‐6 is by stopping the TLR4/NF‐*κ*B signaling path, which is important for making proinflammatory cytokines. Research using a rat model of traumatic brain injury has shown that HBOT strongly blocks the activation of the NF‐*κ*B and TLR4 pathways, lowers the amounts of TNF‐*α*, IL‐6, and IL‐1*β*, and reduces neuron death, which in turn helps improve the rats′ brain function overall [16]. In addition, HBOT also changes the polarity of microglia, which are the main immune cells in the central nervous system. After a traumatic brain injury, HBOT reduces the activity of proinflammatory M1‐type microglia and promotes the activity of anti‐inflammatory M2‐type microglia. This is shown by a lower release of TNF‐*α* and a higher release of TGF‐*β*1 [17].

The DHG treatment was associated with lower TNF‐*α* and IL‐6 levels than the untreated CG; however, the differences between the combination therapy and the individual treatment groups were not statistically significant. Therefore, the present findings suggest a favorable anti‐inflammatory pattern of the combined intervention. The lower proinflammatory cytokine levels provide significant biological benefits, including reducing endothelial activation and adhesion molecule expression, preventing microvascular obstruction, and reducing the risk of tissue hypoxia and organ damage. Thus, therapeutic strategies that target both the parasite and the host immune response have the potential to prevent the progression of malaria to severe forms and systemic complications [18, 19].

The effects of HBOT appear particularly relevant to the present findings. Previous experimental studies using *P*. *berghei* ANKA have shown that hyperbaric oxygen can protect mice from experimental cerebral malaria, reduce peripheral parasitemia, and decrease inflammatory cytokine expression, including TNF‐*α*. HBOT was also associated with reduced sequestration of T lymphocytes in the brain and improved blood‐brain barrier integrity [20]. The effect of HBOT on IL‐6 was also more evident than that of doxycycline. IL‐6 concentrations were significantly lower in the HG than in the CG (*p* = 0.004). Previous studies have shown that HBOT can influence inflammatory signaling, endothelial activation, and hypoxia‐associated pathways. In experimental cerebral malaria, hyperbaric oxygen has been reported to reduce endothelial activation, parasite and leukocyte accumulation in the brain, and hypoxia‐associated HIF‐1*α* signaling [21].

The parasitemia findings also provide an important framework for interpreting the cytokine results. HBOT and combined therapy produced substantially lower parasitemia than doxycycline alone, whereas no significant difference was detected between HBOT and combined therapy. This pattern suggests that HBOT may have contributed substantially to the reduction in parasite burden observed in this model. Previous experimental studies have reported that HBOT can reduce parasite accumulation and improve cerebral microcirculatory conditions in *P*. *berghei* infection. Mechanistically, improved tissue oxygenation may influence endothelial activation and the inflammatory microenvironment rather than acting solely as a direct antiparasitic intervention [20, 21]. Therefore, the lower cytokine levels observed after HBOT may reflect an interaction between altered host inflammatory responses and reduced parasite‐associated stimulation.

The findings of the present study should be interpreted within the broader evolution of malaria management. Recent comprehensive perspectives emphasize that malaria control has progressed from reliance on conventional antimalarial agents toward integrated strategies combining parasite‐directed treatment, vector control, vaccination, surveillance, and approaches addressing host–parasite interactions [22]. Despite major advances, the emergence and spread of antimalarial drug resistance remain important barriers to sustained malaria control. In this context, the present findings are relevant because they suggest that malaria interventions may influence not only parasite burden but also the systemic inflammatory environment. However, the observed reduction in inflammatory cytokines should not be interpreted as evidence that immunomodulation can replace effective antiparasitic therapy. Rather, it supports the concept that future malaria strategies may benefit from combining effective parasite control with carefully regulated modulation of host inflammatory responses. Such an approach is particularly relevant because excessive inflammation can contribute to tissue injury and severe disease, whereas insufficient immune activation may impair parasite clearance [22].

An additional mechanistic consideration is the interaction between oxidative stress and early cellular immunity during malaria infection. Hyperbaric oxygen intrinsically alters tissue oxygen tension and the cellular redox environment; therefore, its immunomodulatory effects may not be independent of oxidative signaling. Recent experimental evidence has demonstrated that superoxide dismutase 3 (SOD3) is involved in regulating early immune responses during malaria infection [23]. A previous study reported that SOD3 was increased during parasite infection and that SOD3 deficiency was associated with reduced parasitemia and improved survival. Their findings further indicated that SOD3 could suppress early cellular immune responses by limiting IL‐2 expression and IFN‐*γ* production by T cells [23]. These observations provide an important conceptual framework for interpreting the present HBOT findings: Changes in oxygen availability and redox signaling may potentially influence the balance between inflammatory activation and cellular immune responses during malaria. Nevertheless, because SOD3 expression, ROS production, IL‐2, IFN‐*γ*, and cellular immune phenotypes were not measured in the present study, a direct involvement of the SOD3 pathway in the effects of HBOT cannot be established. Further studies incorporating redox biomarkers and cellular immune profiling are therefore required to determine whether HBOT modifies malaria‐associated inflammation through these mechanisms.

The immunomodulatory effect observed with doxycycline should also be considered in comparison with other antimalarial agents that have documented effects on host immunity. Artemisinin derivatives provide an important example. Dihydroartemisinin (DHA), the active metabolite of several artemisinin derivatives, has been reported to influence immune cell heterogeneity in experimental malaria. A previous study demonstrated that DHA treatment altered the composition of immune cell populations and promoted phosphorylation of cyclin‐dependent kinases (CDKs) and mitogen‐activated protein kinases (MAPKs), suggesting that DHA can regulate host cellular responses in addition to its direct antiparasitic activity [24]. This mechanism differs from the cytokine pattern observed in the present study, in which doxycycline treatment was associated with reduced TNF‐*α* and IL‐6 concentrations. The comparison suggests that antimalarial‐associated immunomodulation may occur through multiple biological pathways rather than through a single generalized anti‐inflammatory mechanism. Importantly, however, the present study did not directly compare doxycycline with DHA or other artemisinin derivatives. Therefore, no conclusion regarding the relative immunomodulatory efficacy of doxycycline versus artemisinin‐based therapy can be drawn from the present data. Direct comparative studies incorporating cytokine profiles, immune cell phenotyping, and relevant intracellular signaling pathways would be required to establish such differences.

The present findings are particularly relevant because the reduction in TNF‐*α* was accompanied by a reduction in parasitemia. Parasitemia was highest in the untreated CG (mean 38.53%) and progressively lower in the DG (26.00%), HG (17.81%), and combined treatment groups (16.48%). Thus, the lower inflammatory cytokine concentrations cannot be considered independently from the reduction in parasite burden. One plausible interpretation is that reduced parasite‐associated stimulation resulted in less activation of innate immune pathways and consequently lower cytokine production. However, the present study was not designed to determine whether cytokine reduction was primarily a consequence of reduced parasite burden or whether direct immunomodulatory effects of the interventions independently contributed to the observed changes.

This distinction is important for understanding the effect of doxycycline. Previous experimental work in *P*. *berghei* ANKA‐infected mice demonstrated that doxycycline could protect against experimental cerebral malaria through mechanisms extending beyond parasite reduction. Doxycycline‐treated mice showed reduced inflammatory responses in the brain, including lower expression of TNF and IL‐6, together with reduced chemokine expression, T cell infiltration, and tissue‐degrading mediators such as matrix metalloproteinase‐2 and granzyme B. Notably, protection against experimental cerebral malaria could occur even when parasite loads were not markedly different, indicating that doxycycline may influence malaria‐associated pathology through modulation of host inflammatory responses in addition to its antiparasitic activity [25]. This provides a mechanistic context for the lower TNF‐*α* concentration observed in the present DG.

The present study contributes to this framework by demonstrating that doxycycline and HBOT were associated with reductions in selected systemic inflammatory cytokines in *P*. *berghei*–infected mice. However, the different immunomodulatory mechanisms reported for DHA and SOD3‐related pathways highlight that reduction of TNF‐*α* or IL‐6 alone does not fully characterize the host response to malaria. A more comprehensive assessment incorporating parasite burden, inflammatory cytokines, redox status, and immune cell phenotypes will be necessary to determine whether the observed cytokine changes represent beneficial restoration of immune homeostasis or simply a reduction in inflammatory activation.

The limitation of this study is that cytokine assessment was limited to TNF‐*α* and IL‐6 and was performed at a single experimental time point due to financial and funding constraints. Future studies should evaluate a broader panel of proinflammatory and regulatory cytokines at multiple time points to better characterize the temporal dynamics of the immune response and the effects of doxycycline, HBOT, and their combination.

## 5. Conclusion

Both doxycycline and HBOT exert immunomodulatory effects by reducing TNF‐*α* and IL‐6 levels in experimental malaria. The present findings suggest that doxycycline and HBOT may modulate systemic inflammatory responses during experimental malaria. The combination of doxycycline and HBOT shows a favorable anti‐inflammatory profile against malaria‐associated inflammation.

## Funding

No funding was received for this manuscript.

## Conflicts of Interest

The authors declare no conflicts of interest.

## Data Availability

The data that support the findings of this study are available from the corresponding author upon reasonable request.
